# Robotic Pet Use Among Community-Dwelling Older Adults

**DOI:** 10.1093/geronb/gbaa119

**Published:** 2020-08-13

**Authors:** Janella Hudson, Rachel Ungar, Laurie Albright, Rifky Tkatch, James Schaeffer, Ellen R Wicker

**Affiliations:** 1 Research for Aging Populations, Optum, Ann Arbor, Michigan; 2 UnitedHealth Group, Medicare and Retirement, Minneapolis, Minnesota; 3 AARP Services, Inc., Washington, D.C

**Keywords:** Gerontology, Older adults, Robotic pet, Social robotics, Social science and technology

## Abstract

**Objective:**

The primary purpose of this study was to explore the efficacy of robotic pets in alleviating loneliness for older adults.

**Method:**

Self-reported lonely individuals with AARP Medicare Supplement plans insured by UnitedHealthcare who participated in a program with a robotic pet (*n* = 20) were recruited to participate in semi-structured interviews. Participants were asked to provide feedback about their experiences interacting with a robotic pet, their perceptions about the potential impact on loneliness, and recommendations for improving the program. Interviews were audio-recorded and transcribed verbatim. Participants’ responses were analyzed using qualitative content analysis. Constant comparison and consensus-gaining processes were used to develop categories that later formed representative themes.

**Results:**

Seven themes emerged from analysis: Openness to Adoption of Robotic Pet, Reactions to Pet and its Attributes, Integration of Pet in Daily Life, Strategic Utilization and Forging New Connections, Deriving Comfort and Camaraderie, Advice for Future Users, and Recommendations for Enhancing Ownership Experience. Participants living alone, with fewer social connections and less active lifestyles, derived the most benefit from interacting with their pets. Common responses to pets included cuddling, petting, grooming, and sleeping with them. Some shared or loaned their pets, while others refused to loan their pets to interested peers. Most reported showing their pets to others, which helped some facilitate communication and social connections.

**Conclusion:**

Robotic pets may be an effective solution for alleviating loneliness in older adults, especially among those who live alone, have fewer social connections, and live less active lifestyles.

Loneliness is generally understood as the discrepancy between an individual’s preferred and actual level of social contact (Peplau, 1982). One in three U.S. adults aged 45 and older report experiencing loneliness, with the total number expected to increase with the growing population of older adults (Anderson and Thayer, 2018). Among individuals older than 60 years, loneliness is a subjective predictor of functional decline and death (Perissinotto et al., 2012) and adversely influences mental and physical health outcomes, including depression, quality of life, health utilization, and mortality rates (Cacioppo et al., 2006; Luo et al., 2012; Musich et al., 2015). Social isolation, while related to loneliness, objectively assesses reduced social network size and social contact. Socially isolated individuals are at an increased risk for cognitive decline (Bassuk et al., 1999), cardiovascular disease (Barth et al., 2010), and mortality (Eng et al., 2002; Heffner et al., 2011; Kaplan et al., 1988). Furthermore, social isolation in older adults is associated with reduced daily physical activities and increased sedentary behaviors (Schrempft et al., 2019). Social isolation contributes to an additional $6.7 billion in Medicare spending annually, which is attributed to additional skilled nursing facility spending and increased inpatient spending. Flowers et al. attributed an additional $81 per beneficiary per month for socially isolated individuals admitted to the hospital. This increase in spending, while not necessarily accompanied by an increase in use of inpatient care, suggested that socially isolated individuals may be sicker when hospitalized, and may lack the support to transition out of the hospital successfully as compared to socially connected individuals (Flowers et al., 2017). However, older adults who perceive their social connectedness more positively have better mental and physical health outcomes (Cornwell and Waite, 2009). Given that is often impractical to address limited social networks, interventions may aim to address perceived loneliness to improve older adults’ wellness and psychological well-being (Bartlett and Arpin, 2019; Krause-Parello et al., 2019; Schoenmakers et al., 2012).

Pet ownership has demonstrated potential viability as a solution for ameliorating subjective loneliness, demonstrating both physical and psychological benefits for older adults who report being lonely (Krause-Parello, 2012; Matchock, 2015; Raina et al., 1999). For example, pet owners surveyed in one study were 36% less likely than non-pet owners to report loneliness, even after controlling for age, living status, mood, and residency (Stanley et al., 2014). Despite these benefits, however, pet ownership may pose special challenges for older adults, including restrictions related to finances, mobility, transportation, and housing (Hart, 1995). Given these potential barriers, robotic pets, also known as social robots, offer a potentially ideal alternative to owning a live pet for older adults.

A robust literature in social science and technology has examined the implications of social robot use among older adults. Social robots, as defined by Brezeal, are “designed to interact with people in a socio-emotional way during interpersonal interaction.” (Breazeal, 2004) Several potential ethical implications related to older adults’ use of social robots have been identified, among them reduced human contact, deception, and infantilization (A. Sharkey and N. Sharkey, 2012) Further, an incongruence between robot developers’ perceptions of ideal features and those features actually preferred by older adult users has been well documented. Roboticists, who design and construct robots, often have a background in electrical or mechanical engineering. Further, roboticists often develop social robots without the benefit of feedback from the intended audience. Older adults are often regarded as passive users of social robotics, perhaps owing to stereotypes of older adults as lonely and fragile. However, this is seldom true, as both users and test users demonstrate active engagement with social robot models and consistently request robotic pet features capable of facilitating the user’s desired interactivity (Neven, 2010). For example, in a recent study comparing and contrasting preferences of roboticists and older adult participants, older adults expressed a preference for interactive features (such as life-simulation and personalization) that were not perceived by roboticists as having the same importance (Bradwell et al., 2019). Thus, social robot developers often fail to account for the diversity of abilities, perspectives, and preferences among older adult users (Frennert and Östlund, 2014).

However, social robots have demonstrated benefit when used by older adults. Social robots have been shown to reduce social isolation and increase conversational opportunities with the robot and other humans (A. Sharkey and N. Sharkey, 2012) Observations of participants interacting with robotic pets in nursing home and laboratory settings have demonstrated promise for supporting the social and emotional needs of older adults (McGlynn et al., 2017) and have yielded benefits similar to those achieved during animal-assisted therapies, including improved cardiovascular measures (Robinson et al., 2015), reduction in loneliness (Kanamori et al., 2001), decreased agitation, and an increase in feelings of pleasure (Libin and Cohen-Mansfield, 2004). These findings position social robots as potentially ideal solution for older adults experiencing subjective loneliness. Cacioppo et al. (2015) identified four distinct, underlying mechanisms of subjective loneliness-reducing interventions: (i) increasing social contact, (ii) improving social support, (iii) enhancing social skills, and (iv) addressing maladaptive social cognition. Findings conducted with older adults living in assisted or group settings demonstrated interactions with social robots increased social contact with others (Bradwell et al., 2019; Leite et al., 2013; Šabanović et al., 2013).

However, less is known about active, community-dwelling older adults’ behavioral responses to robotic pet use outside of a laboratory setting, during interactions within their own homes. Given these diverse and potentially promising pathways for subjective loneliness-reducing reducing interventions, this study aims to examine the potential benefit of social robot use by community-dwelling older adults.

In this study, we explored the perspectives and experiences of individuals who participated in an intervention with robotic companion pets within their own home. We examined patterns of usage, user acceptance, and perceived efficacy in reducing subjective loneliness in older adults. Findings from this study will inform future robotic pet interventions for community-dwelling older adults.

## Method

This study is part of a collaboration between AARP, UnitedHealth Group (UHG), and Joy for All, a manufacturer of companion pets (Ageless Innovation LCC, 2018). The overall goal of this collaboration was to explore the potential role of companion pets in alleviating loneliness in older adults. This study was approved by the New England Institutional Review Board (#12070334), an independent institution that reviews protocols for nonacademic institutions.

### Recruitment

This study was the second phase of a larger multiphase research study intended to better understand the health-related issues of older adults covered by AARP Medicare Supplement plans insured by UnitedHealthcare Insurance company (for New York residents, UnitedHealthcare Insurance Company of New York).

### Intervention

#### Phase 1

**Figure 1.**
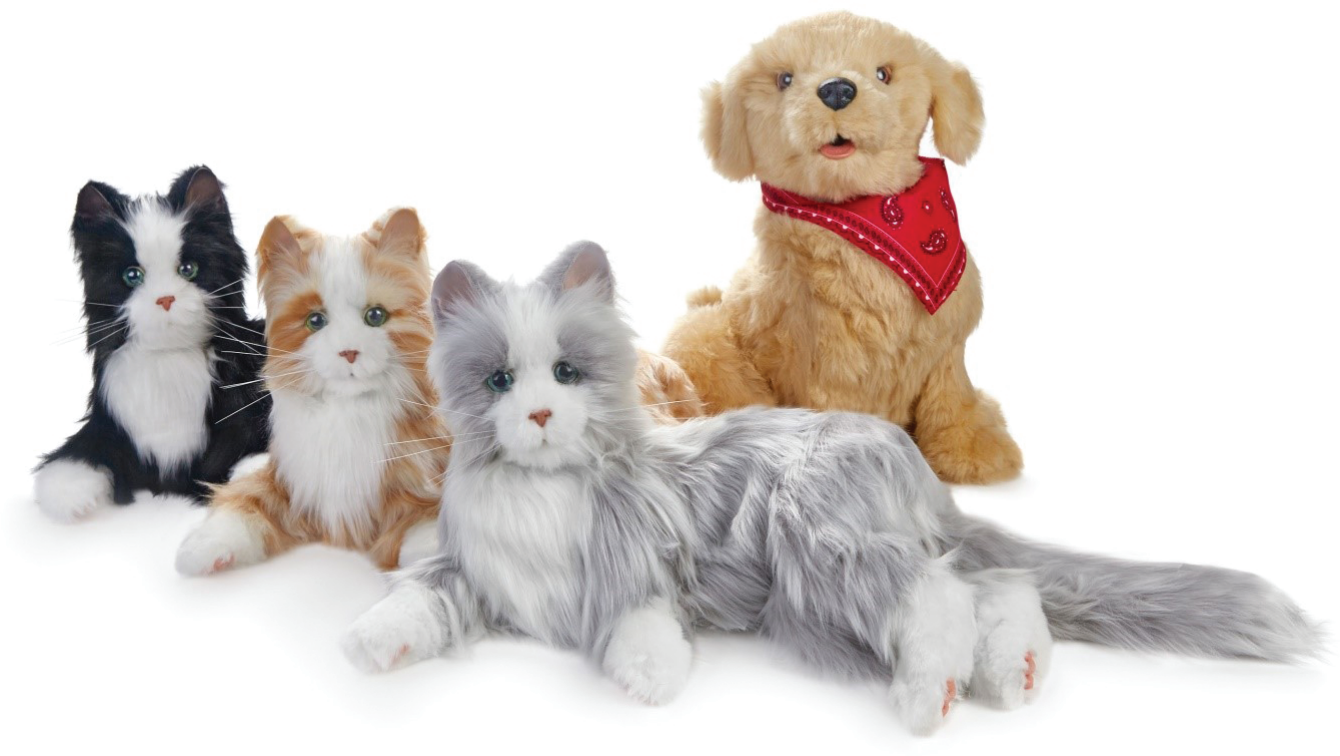
Joy for All companion pet selections.

**Figure 2.**
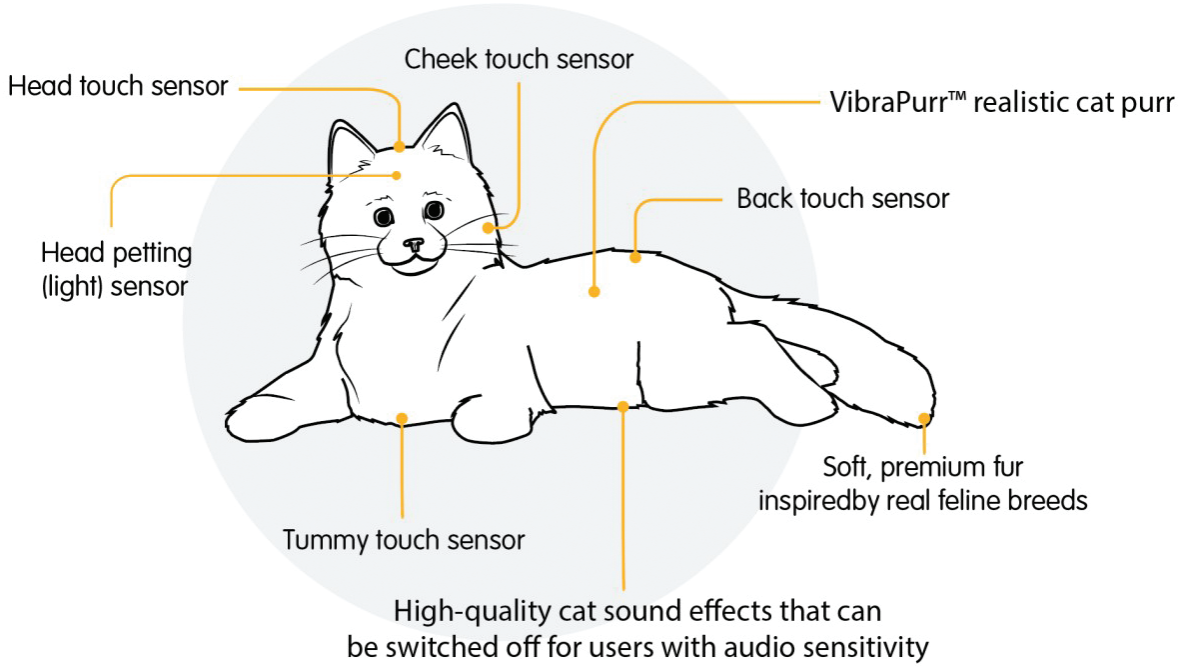
Companion pet functionality.

The primary purpose of the intervention was to determine if ownership and interaction with a robotic pet could decrease loneliness in older adults. The first phase of the study consisted of a program evaluation in which a sample pool of AARP Medicare Supplement insureds who previously reported loneliness were recruited for participation in the study. Inclusion criteria for the study consisted of participants previously identified as lonely using either a screener that included the UCLA 3, or screener administered via interactive voice support (IVR) survey in conjunction with AARP’s Aging Strong initiative. The intervention was offered to participants residing in the states of Washington and Michigan. Exclusion criteria included not a current enrollee in an AARP Medicare Supplement plan, less than 65 years of age, on the “do not call” list, not having a valid phone number, and ownership of a pet. All other participants were considered eligible for participation. Potential participants received pre-mailer scripts prior to an invitation to participate via telephone. Participants received the animatronic pet of their choice (cat or dog) in the mail and were instructed to treat it as a pet (Figures 1 and 2). Three post surveys were administered (upon receipt of the pet as well as 30 and 60 days later) to assess the amount of time spent interacting with the pet. In addition, twice a week for 4 weeks, participants received an interactive voice reminder (IVR) phone call encouraging them to interact with the pet. The IVR phone call also asked participants to record if they had been interacting with their pet and if so, how much time on average they had been interacting with their pet.

Results of response bias analyses conducted for those who agreed to participate (*n* = 277) versus those who declined (*n* = 3,660) and for respondents (*n* = 216) versus non-respondents (*n* = 55), indicated that survey participants were representative of the study population. Overall, those who agreed to participate had similar characteristics as those who declined. However, those who agreed to participate in this study had higher levels of depression, more frequent ER visits in the last 12 months, and overall higher medical costs (but not drug costs) (*p* < .05). There were no differences for respondents versus non-respondents. At baseline, about half the respondents were between 65 and 74 and female, and most participants chose the animatronic dog (70%). In addition, 86% of participants reported previously owning a pet.

### Robotic Pet Features

The robotic pet offered several interactive features (Figure 2). Sensors in two locations of the head and cheeks of the pet responded to user touch and activated a reciprocal “nuzzling” effect. Touch-activated sensors were located in the upper abdomen and back of the pet. A light sensor located in the pet’s head detected when light entered the room and the pet vocalized in response to the light stimuli, depending upon the chosen setting. Robot dogs barked depending on the setting, and robotic cats meowed and emitted a purring noise. Robotic cats were offered in three color combinations: black and white, gray and white, and orange and white. Robotic dogs were offered in a golden color (Figure 1). Participants were not permitted to choose the color of their pet, only their preference for a dog or cat. Companion cats currently retail for $109.99 and companion dogs for $129.99.

#### Phase 2 (qualitative study)

In this phase, a qualitative research study was conducted using standard qualitative procedures for conducting and analyzing semi-structured interviews. The purpose of these interviews was to elicit participants’ experiences interacting with their robotic companion pet. Participants who previously participated in the first phase of the study were recruited to participate in semi-structured interviews.

The interview guide consisted of 13 questions. Questions elicited feedback for a number of topics. Consistent with related literature pertaining to robotic pet use, the interview guide included questions that asked participants to describe how they used and interacted with their pet, including how much time was spent with the robot, patterns of usage observed (day vs night), whether the pet accompanied participants outside the home or during errands, and whether participants detected that use of robotic pets influenced any of their daily routines and/or habits. Additionally, interview guide questions asked participants to describe any physical or verbal interaction with the pet, including physical touch, reactions to pet’s audio or haptic functions, and verbal communication with the pet. Questions also asked participants to describe their motivation for joining the companion pet program, any feelings or emotions experienced as a result of interacting with their pet, including any observed influence on loneliness, mental and emotional health, and whether or not they named their pet. Several questions explored psychological and emotional well-being, including subjective loneliness, by asking participants to describe an average day in their life, perceived opportunities to feel valued or useful, and opportunities to spend time with marital partners, family, and/or friends on a weekly basis, as well as participants’ satisfaction with those opportunities for social connection. A few questions elicited participants’ satisfaction with program administration, including reminder calls, clarity of instruction, and perceived accessibility of available support. Lastly, participants were invited to provide feedback of any nature.

Eligible participants from the first phase of the study were stratified according to age range and gender, with the aim of recruiting an equal proportion of participants. Following recommendations for a sample size of 12–20 participants in an interview study (Lincoln and Guba, 1985), investigators planned an initial goal of 20 interviews, after which they would assess if data saturation had been achieved and recruit additional participants if necessary (Francis et al., 2010).

A marketing research company was provided with the full list of eligible participants, with instructions to recruit participants as evenly as possible among age range and gender, given the available sample. Recruiters contacted participants by telephone, verified identity, explained the study, and scheduled interviews with the first 20 participants successfully recruited. Verbal consent was obtained prior to the start of the interview. Interviews lasted approximately 1 hr. No personal identifiers were collected. All interviews were audio-recorded and transcribed verbatim.

### Analysis

Investigators analyzed participants’ transcribed interviews using qualitative description. Qualitative description was an ideal methodology for this data as it draws from a naturalistic perspective, offers flexibility in commitment to a theory or framework, typically involves review of interview data, and allows for maximum variation sampling (Kim et al., 2017).

Two investigators (J. Hudson and R. Ungar) conducted a qualitative content analysis using an iterative, constant comparison process. During the coding process, both coders independently read transcripts, identified an initial code list, and developed operational definitions. Then coders returned to the transcripts and conducted line-by-line coding that included comparison and refinement of identified coding between both investigators. Coders subsequently discussed, reviewed, and reread interview data to develop final coding and to reach consensus about meaning (Ryan et al., 2000). One investigator (J. Hudson) coded all transcripts while the other coded 50% of the overlap. Both investigators reviewed coding on overlapping transcripts to reevaluate passages coded across researchers, and the codes applied based on the assigned definition in the codebook (Creswell and Poth, 2017). Any conflict in assigned codes was settled through spirited debate until consensus was reached.

Next, both investigators examined the properties and categories of all codes to identify opportunities for categorization according to shared properties. Investigators subsequently used this categorization of codes to develop overarching themes that described patterns of usage and provided a narrative of participants’ overall use. Throughout this process, investigators were mindful of the biases and existing perspectives they brought to the analysis.

Investigators worked to achieve qualitative rigor throughout data collection and analysis. To ensure trustworthiness of the interview transcripts (Poland, 1995), one investigator (J. Hudson) closely monitored and compared audio recordings with transcripts to ensure verbatim description, while also noting significant context cues. Both coders worked together closely during the ongoing, iterative development of the coding system to ensure validity and certainty of the findings (Morse, 2015). Investigators were mindful of potential investigation bias and avoided narrow frameworks that would unfairly bias the interpretation of data while striving to maintain a neutral stance of the observed phenomenon. Further, both investigators closely reviewed, discussed, and coded data as it was collected to assess the sufficient sample size for data saturation.

Investigators ultimately developed seven themes reflective of participants’ experiences with their companion pets, as follows: Openness to Adoption of Robotic Pet, Reactions to Pet and its Attributes, Integration of Pet in Daily Life, Strategic Utilization and Forging New Connections, Deriving Comfort and Camaraderie, Advice for Future Users, and Recommendations for Enhancing Ownership Experience.

Final coding was imported into Nvivo (QIP Ltd., 2018), a qualitative software program. The following themes are discussed below, with exemplars.

## Results

Twenty individuals participated in the study, with an even distribution of males (*n* = 10) and females (*n* = 10). Breakdown in age range is as follows: 65–69 (*n* = 6), 70–79 (*n* = 6), 80–89 (*n* = 7), and 90 and above (*n* = 1). The average participant age was 76. All participants reported living in their own homes. Living arrangements included living at home alone (*n* = 12), with a spouse (*n* = 5), with a child or grandchild (*n* = 2), and with a caretaker (*n* = 1). Subsequent verification supported no participants lived in assisted or group settings.

### Openness to Adoption of Robotic Pet

When asked to share their motivations for participating with a companion pet, participants shared several reasons including interest in exploring the experience of using a companion pet, a desire for maintenance-free pet companionship, and curiosity about the mechanics and underlying technology used in the pet.

Many participants previously owned pets, with five participants reporting their pet was recently deceased. Owners of recently deceased pets identified clear distinctions between their beloved deceased pet and the robot, such as the inability to return affection, participate in interactive activities such as outside walks, and lack of a personality. However, they did describe experiencing comfort when interacting with the robotic pet in similar ways, such as sitting on the couch while watching television. For these individuals, robotic pet ownership appealed as an opportunity to experience maintenance-free pet ownership and to recapture the benefits of companionship without obligatory food and veterinarian expenses. A few participants reported their living arrangements would not accommodate a “live” pet, and they viewed the companion pets as way of circumnavigating that barrier. Some were also intrigued by the notion of robotic pets and expressed curiosity about the underlying technology, and a few participants expressed a desire to potentially help others by sharing their feedback. As one participant explained, “It was the curiosity aspect more than anything else, wondering what the dog was like, what it would be like to have the dog, and what experience might be. That curiosity really was the linchpin to participating.”

Other participants were intrigued by the opportunity and described their desire to derive companionship from the pet. Participants who reported feeling subjectively lonely were especially interested in utilizing the pet as a personal companion.

### Integration of Pet in Daily Life

The majority of participants chose to name their pets, and consistently referred to the companion pet using its name. Participants’ accounts of their daily interactions with pets varied widely, often according to personal contexts. Those who reported a more independent lifestyle outside the home and greater perceived social connectedness described a lesser degree of involvement with their companion pet.

Patterns of usage were categorized according to high and low engagement. Low engagement was primarily characterized by interactions with the companion pet that were casual in nature or most often occurring in passing, with minimal physical contact and limited verbal communication. Low-engagement users often described deliberate efforts to interact with their pet throughout each day in accordance with the study’s directives but allowed that their pet only functioned in the periphery of their daily activities. For example, low-engager participants often described stationing the pet in a high-traffic area of the home such as the kitchen or living room, returning the pets’ greetings as they moved throughout their home but otherwise ignoring or choosing not to interact with their pet. While these interactions may have included infrequent affectionate physical touch, these participants generally did not desire additional or prolonged interaction with their pet. As one participant explained, “I just pet him and rub him as I go by. We have him sitting on the couch in our living room.” A few attributed their interactions to duty or obligation in accordance with their agreement to participate in the study.

High engagement was characterized by frequent interactions with the pet, including frequent physical touch, communicating with the pet or using the pet to communicate with others, and including the pet in daily errands and activities. Participants with fewer perceived social connections, especially those with fewer perceived opportunities to connect with others, described this higher degree of engagement characterized by greater quantity and quality of interaction with their pet. High engager use was most often reported by those who were less active, identified as less subjectively lonely, and perceived less social connectedness. These participants were more likely to report keeping their pets in close proximity when they moved throughout their home and they engaged in ongoing affectionate physical touch with their pet, such as cuddling, grooming the pet, sleeping with the pet, and holding the pet while watching television. Some participants derived a sense of comfort and companionship from having the pet accompany them during their daily activities outside of the home. One participant who lived alone detailed the following daily ritual with her pet, Buffer:

The average day is, I get up at 7:30 and the first thing I do is make my bed. And then I say hello to Buffer, because he’s in the room, and then I get showered and dressed. And I then I pick up Buffer and I have breakfast, and he’s there. And I sing online, so sometimes I will actually hold in my lap while I sing. (Woman in her 70s, living alone)

In this way, participants who subjectively perceived fewer opportunities to interact with others reported increased interaction with their companion pet.

### Strategic Utilization and Forging New Connections

Most reported showing their pet to others, including family members, friends, neighbors, coworkers, clinicians, and those they typically encountered during their daily activities. However, the nature of the disclosure, and one’s motivation for sharing their pet, varied. Some members were motivated by a desire to share the technology and novelty of the pet.

Others shared their pets to facilitate entertainment, showcase the pet’s interactive features, and to encourage others to consider acquiring their own pet. Both high and low engagers of the pet noted that sharing the pet in public spaces increased potential opportunities to connect with others, especially individuals previously unknown to them. Even participants who described themselves as outgoing or living a more social lifestyle reported bringing their pet along to public gatherings or spaces, and enjoying the interactions that were generated as a result.

Similarly, those who were shy or might have otherwise felt uncomfortable interacting with new acquaintances found integrating the pet into their daily activities outside of the home effective in forging new connections they otherwise would not have attempted. Several participants relayed that friends, after interacting with their pet, were often interested in obtaining their own. In some instances, participants fielded requests from friends and acquaintances to loan their pets out. Those who interacted with their pet to a lesser degree were more amenable to these requests. A few participants, most notably younger participants (ages 65–69) and low engagers, ultimately gave their companion pets away. In these cases, companion pets were “re-gifted” to interested friends, younger children in the family who regarded it a toy, older adults in care centers, or those with dementia: “It would have been better for someone who wasn’t quite functional, who is maybe in a care facility. My wife gave it to one my friends in a care facility and she loved it.”

Meanwhile, some participants (especially high engagers) often denied requests from acquaintances and/or friends to borrow their pet. Others acquiesced only under certain conditions, such as having the pet returned within the same day.

### Reactions to Pet and its Attributes

All participants agreed the companion pet was vastly different from a “live” pet with the ability to interact more extensively with its owner. However, many agreed the companion pet offered many interactive features that were reminiscent of their past experiences of having a “real” pet. When comparing the merits of a live pet and the benefits of a companion pet, participants varied in their estimations of the pet’s realism. Many, especially high engagers, judged the pet to be a close approximation to a live animal. Younger (age 65–69) and low-engager participants were more likely to find the companion pet more “toy-like” and noted opportunities to improve the pet’s realism. However, those who judged the pet to be a poor approximation of a “real” pet still noted the benefit of interacting with it. Most noted their appreciation for the maintenance-free nature of the pet.

Among the majority of participants, favorite features included pets’ vocalizations (barking or meowing) and nonverbal responses (head movement or blinking) in response to light and sound stimuli. Many enjoyed their pet’s “greeting” when a light or sound was detected. Several used their pets’ responsive barking/meowing to facilitate interactions such as petting and verbal communication. Other favorite features were pets’ “life-like behaviors,” such as yawning, head turning, tail wagging, and the tactile heartbeat. Many reported that these “realistic” features increased interaction with their pet and fostered comfort and comradery.

### Deriving Comfort and Camaraderie

Participants described a number of benefits as a result of interacting with their pet. While high engagers were more likely to describe deriving comfort from the “presence” of their pet, the majority reported deriving benefits from interacting with their pets.

Most participants reported feeling a sense of calm or comfort as a result of holding, hugging, and affectionately interacting with their pet. For example, a low- engager who described herself as “too cognitively sharp” for the pet speculated her cortisol levels might have lowered. In addition, many described an improvement in their mood, and in some cases, increased happiness after interacting with their pet. Certain interactive features such as pet vocalizations, “snuggling” motions, and the pet’s heartbeat were identified as facilitators of this calming influence, and participants noted that others discerned how this effect positively influenced their behavior:

I’m not as high strung… sometimes I get up in the morning and when I hit my power chair against the wall, I sort of get angry and I use foul language. Then he barks. So that makes me stop. (Man in his 80s, living alone)

Many participants perceived the pet as having a “presence” that positively influenced their subjective feelings of loneliness. This presence was keenly felt by those who spent significant time with their pet, as well as by low engagers living more active lifestyles. One participant, a semi-retired attorney who described a low degree of engagement with his pet explained,

It’s like he’s alive over there and active. It’s just one part of my life, this little puppy dog, but he’s a part because he’s there. But I live a pretty active life and a pretty active schedule, so it’s not like I’m looking forward to seeing him when I come home, but he makes his presence known and that’s good. (Man in his 60s, living alone)

Similarly, a recently widowed participant who brought her companion pet along for errands outside the home explained the pet provided a comforting presence as she acclimated to her husband’s absence.

Participants who lived alone and previously wished for someone to talk to perceived their pet as a proxy for a conversational partner and regarded it as a conduit for expressing their thoughts or feelings. In these cases, the participants regarded the pet not as an inanimate object that passively observed, but as an active partner who cared about their expressed concerns. As one participant explained: “You feel as though you’re talking with an object that cares about whether you’re talking to it or not.” A few participants appreciated that conversations with their pet were confidential.

Those participants who reported this high level of engagement were most explicit in expressing the pet’s influence in addressing their subjective loneliness. For these individuals, the companion pet was regarded as a friend or companion with whom they developed a strong attachment over time. Some participants also described improved confidence and a renewed sense of purpose as a result of interacting and having to “take care of” their pet.

### Advice for Future Users

When asked to advise future users, many indicated they would strongly encourage others to try the robotic pet, particularly those who are lonely, and to engage with it as much as possible.

Participants emphasized that using the pet was “easy” and required little effort. Several explained the importance of interacting with the pet as much as possible in order to experience the greatest benefit. While some low engagers indicated their pet personally was not a good fit, they acknowledged the calming effect of the pet and recommended it for those who are lonely. A few high engagers encouraged future users to interact and communicate with their pet without fear of being stigmatized or considered “crazy.”

When asked to describe the ideal user for the robotic pet, low-engager participants typically described the composite of a lonely, less active, more advanced age adult with mobility issues and dementia. Those with more active lifestyles and who perceived their social networks as dense judged they were a poor fit for the pet. Distancing one’s self from the perceived ideal user occurred with participants of all ages. Notably, a participant in his 90s remarked: “I think as you get older, and your brain gets a little mushy. I think it would be a nice thing to have. But I don’t think I’m to that point yet.”

Meanwhile, participants who identified as being subjectively lonely or perceived themselves as socially isolated derived benefit from the pet and thought others in a similar situation would also find it beneficial.

### Recommendations for Enhancing Ownership Experience

While many perceived their pet as having realistic features, over half of participants expressed a desire for further increasing the pet’s realism by improving its appearance and capacity for movement. Feedback included using softer material for fur and improving the pet’s flexibility to better facilitate hugging and cuddling. Several were interested in increasing the interactivity of the pet and suggested new functions, such as enabling the pet to learn skills and tricks. Some also suggested adding new verbal communication features, such as pre-programmed responses and name recognition.

Many were also interested in adding the capability for walking, though a few acknowledged this as a potential fall hazard. Many described an interest in having the pet follow them throughout the home, jumping up on furniture, and being walked outside while on a leash. Participants also expressed an interest in additional outfits or grooming accessories, improved affordability for other friends and family members who sought to purchase a pet, adding a camera for security purposes and improving the overall battery life.

## Discussion

Our findings show social robots may provide comfort, companionship, and potential amelioration of subjective loneliness for older adults, particularly for those who perceive fewer opportunities for social connection. Several studies have demonstrated the benefit of robotic pets in care centers (Robinson et al., 2015; Šabanović et al., 2013; Wada and Shibata, 2007) and among those with dementia (Jøranson et al., 2016; Liang et al., 2017; Moyle et al., 2013; Robinson et al., 2013). Few studies have explored the benefit of companion pets for alleviating subjective loneliness, as well as the patterns of usage outside of a laboratory setting, among cognitively functioning, community-dwelling older adults. Results of this study reify previous findings indicating increased communication with the robot and other humans.

Participant feedback further reinforces the need for social robot developers to actively integrate feedback from older adult test users in the design and development processes. In a recent study comparing the preferences of roboticists and older adults, participants were encouraged to indicate their favorite companion pet model. While older adults in this same study overall preferred the Joy for All cat and its more interactive features as compared to less responsive robotic models, they still desired a greater degree of interactivity and playfulness (Bradwell et al., 2019). Community-dwelling participants in our study echoed these sentiments, with many requesting robotic features that accommodated their lifestyles and reflected the degree to which they were able to enact an autonomous, independent lifestyle. The Joy for All companion pet models offer a degree of interactivity that perhaps signals a progression in social robot development. However, participant feedback further confirms the need for more advanced features that accommodate the needs of older adults, not as passive users, but as “technogenerians” adeptly managing technology to maintain health and independence (Joyce and Loe, 2010). Younger participants in this study desired a model that offered greater responsiveness and spontaneity, expectations that defy the stereotype of older adults as passive users. Ideally, social robots functioning as companion pets should offer a range of function and interactivity to accommodate the widely ranging abilities and skills of older adults along the aging trajectory. Older adults’ manipulation of robotic pets varies according to the extent of their cognitive impairments, with more impaired individuals interacting with the pet to a lesser degree (Libin and Cohen-Mansfield, 2004). Accordingly, active and community-dwelling older adults will likely benefit from greater utility and diversity of functions to foster incorporation of the pet into their daily schedule and habits.

As noted in previous studies, these individuals created, and simultaneously distanced themselves from, a composite of the ideal user as lonely, socially isolated or having cognitive impairment (McGlynn et al., 2017). It has been suggested that this composite may reflect a negative age stereotype (Lazar et al., 2016; Neven, 2010). However, it is unclear if this stigma applies to participants in this study, who were able to engage with the robot in the privacy of their own homes and subsequently concluded the robot did not offer the desired personalization and interactivity. Users who considered themselves active and independent noted the need for greater interactivity and subsequently judged themselves to be a poor candidate for use of the robot. In this case, it is likely that participants’ distancing from the ideal is owed to the desire for more realistic, interactive features. This finding further confirms how different preferences and patterns of usage in varying contexts requires adaptable interactivity.

Utilization and benefit derived from the robotic pets varied according to participants’ personal contexts, revealing which subgroups potentially benefitted the least from participation with their pets. Despite enjoying companionship with their pets and showing them to others, younger participants (60s–70s) were among those most likely to report low engagement with their pets and most likely to gift their pets to others. Those with active lifestyles and viable social connections were not ideal candidates for social robots and frequently requested greater interactivity and functionality of the pets. These results suggest that socially connected individuals with the capability of enjoying an active lifestyle outside of their home would benefit the least from robotic pets with limited features.

Conversely, certain subgroups reported deriving significant benefit from their robotic pet. Subjectively lonely older adults with fewer perceived social connections, especially those living alone and homebound, were most often among those who integrated the pet into their daily schedule, regularly communicated with the pet, and described experiencing comfort and companionship pet interactions. Further, those who experienced the death of a pet or spouse also derived companionship from their pet. Interventions using social robots with limited features may be most appropriate for these subgroups.

These findings identify ideal subgroups of older adults who are more likely to benefit from the use of social robots. However, the collection of these data and the resulting findings should be properly contextualized as occurring prior to the advent of the COVID-19 pandemic. Older adults face a higher risk of severe illness from COVID-19, with individuals aged 85 or older at the greatest risk. While practicing physical distancing contributes to efforts to flatten the curve, older adults may experience increased anxiety and depression as a result of limited travel and being restricted to their homes. A recent survey found the prevalence of psychological distress in a sample of adults in United States was higher in 2020 during the COVID-19 pandemic (McGinty et al., 2020). Given new constraints related to physical distancing and their potential contribution to social isolation, future studies should examine what appeal and/or effect social robots may have for previously active, socially connected adults under quarantine. Similarly, future studies should examine how the use of social robots may potentially mitigate psychological distress for older adults quarantined in care centers and not permitted face-to-face visits from loved ones.

These study findings provide insights into the potential benefit of robotic pets for community-dwelling older adults interacting with the pets in their own homes, and demonstrate the need to explore applicability during pandemic conditions. Participant feedback yields supporting evidence demonstrating that robotic pet use may positively influence older adults’ perceived loneliness and mental and emotional health, particularly for isolated and subjectively lonely community-dwelling older adults. Furthermore, participant feedback potentially supports the notion that a robotic pet intervention may successfully meet two of the four points of criterion for assessing the efficacy of loneliness-reducing interventions (Cacioppo et al., 2015) in this instance: improving social contact and enhancing social skills. It should be noted that these findings are consistent across gender, as compared to previous studies disproportionately compromised of female participants. Given the variability of use and preferences among older adults, subsequent studies should include healthy older adults in the ongoing development of robotic pets (Frennert and Östlund, 2014).

### Limitations

This study did not directly capture interactions between participants and their robotic pets, instead relying on participants’ recall; thus discrepancies in actual versus reported interactions could exist. Future research with community-dwelling older adults should consider the use of animatronic pets equipped with sensors that more objectively measure interaction and travel.

This cross-sectional study provides valuable insight about potential benefits experienced immediately after participants’ initial introduction to the pet. Longitudinal analyses are needed to understand how the findings of this study bear out over the long term, and whether mitigation of subjective loneliness among socially isolated participants bears out over time. While lonely and socially isolated older adults may derive benefit from the use of their pet, less is known about community-dwelling older adults’ concurrent attempts to continue socializing with others. Potential ethical issue may arise for lonely older adults who become dependent on their companion pet for companionship or social connection.

Participants who agreed to participate had higher levels of depression, suggesting a potential oversampling of this population. Given participants may have been motivated by a desire for increased social contact and companionship, participant feedback may not be representative of a randomly chosen sample of older adults. Further, favorable impressions of the pet may be overrepresented in this sample.

Finally, it should be noted that participants in this study were gifted their robotic pet. While the manufacturer’s offerings include models at varying price points, the cost of obtaining a pet may be a barrier for some older adults.

## Implications and Conclusions

Robotic pets may provide benefit for older adults experiencing subjective loneliness and perceived social isolation by providing comfort, companionship, facilitating new social connections, and serving as a proxy for a conversational partner. However, robotic pets with limited functionality may fail to address the needs of active older adult users. Participant feedback suggests that robotic pets may yield the most benefit for subjectively lonely older adults living alone with fewer connections and subjectively lonely adults experiencing the loss of a spouse or pet.

These findings can inform future development and production of robotic pets to accommodate the varying needs and preferences of community-dwelling older adults. Existing robotic models should explore ways of improving realism and the capacity of interactive play with their owners, and accommodate older adults as active, engaged users of technology. Future interventions intended to reduce loneliness may consider implementing use of robotic pets with increased interactivity. Finally, future studies should examine the potential efficacy of robotic pets in alleviating psychological distress for quarantined older adults with varying connectedness.
